# Evidence for a Mass Dependent Step-Change in the Scaling of Efficiency in Terrestrial Locomotion

**DOI:** 10.1371/journal.pone.0006927

**Published:** 2009-09-07

**Authors:** Robert L. Nudds, Jonathan R. Codd, William I. Sellers

**Affiliations:** Faculty of Life Sciences, University of Manchester, Manchester, United Kingdom; Universidad Europea de Madrid, Spain

## Abstract

A reanalysis of existing data suggests that the established tenet of increasing efficiency of transport with body size in terrestrial locomotion requires re-evaluation. Here, the statistical model that described the data best indicated a dichotomy between the data for small (<1 kg) and large animals (>1 kg). Within and between these two size groups there was no detectable difference in the scaling exponents (slopes) relating metabolic (*E*_met_) and mechanical costs (*E*_mech, CM_) of locomotion to body mass (*M*_b_). Therefore, no scaling of efficiency (*E*_mech, CM_/*E*_met_) with *M*_b_ was evident within each size group. Small animals, however, appeared to be generally less efficient than larger animals (7% and 26% respectively). Consequently, it is possible that the relationship between efficiency and *M*_b_ is not continuous, but, rather, involves a step-change. This step-change in the efficiency of locomotion mirrors previous findings suggesting a postural cause for an apparent size dichotomy in the relationship between *E*_met_ and *M*_b_. Currently data for *E*_mech, CM_ is lacking, but the relationship between efficiency in terrestrial locomotion and *M*_b_ is likely to be determined by posture and kinematics rather than body size alone. Hence, scaling of efficiency is likely to be more complex than a simple linear relationship across body sizes. A homogenous study of the mechanical cost of terrestrial locomotion across a broad range of species, body sizes, and importantly locomotor postures is a priority for future research.

## Introduction

That small terrestrial locomotors are less efficient than larger animals is an established precept within animal biomechanics [1]–[4]. Heglund *et al.* [3] qualitatively and later Full [2] using regression analysis concluded that there was a dramatic decrease in the mass-specific metabolic energy cost of locomotion (*E*_met_) with increasing body mass (*M*_b_) in animals. Furthermore, the mass-specific mechanical work performed on the centre of mass *E*_mech, CM_ was constant across body sizes. Scaling exponents for the two energy forms were *E*_met_ ∝ *M*_b_^−0.32^ and *E*_mech, CM_ ∝ *M*_b_^−0.01^, which indicated an efficiency of transport (*E*_mech, CM_/*E*_met_) range from 0.6% for the smallest animals to 41.4% for the largest animals [1], [2]. Of course, *E*_mech, CM_ does not include a measurement of total mechanical energy – for example it ignores internal sources – but crucially the tenet of body size dependent scaling of efficiency is based upon this measure [1], [2].

Explanations for less efficient locomotion in smaller terrestrial organisms have centred upon elastic storage mechanisms and the size dependent efficiency of muscle [1], but so far empirical evidence for any mechanism is lacking. Recent work, however, has started to look in more detail at the relationship between the cost of transport and body size and has suggested that the relationship between *E*_met_ and *M*_b_ differs between large posturally erect (>1 kg) and small crouched (<1 kg) animals: *E*_met_ scaling as *M*_b_^−0.38^ and *M*_b_^−0.16^ respectively [5]. Intuitively, posture is more likely to influence locomotor efficiency than body size, because, for example, energy savings from spring and pendular mechanisms are negligible in small crouched animals [5]. Consequently, regression analyses of locomotor costs across the entire range of animal sizes are likely to be misleading. Surprisingly, although hypothesised for *E*_met_ [5], a similar size dichotomy in *E*_mech, CM_ has not been considered.

Close scrutiny of previous presentations of existing *E*_mech, CM_ data [2], [3], [6], [7] highlighted two unresolved issues: Firstly, a qualitative division of data into two size clusters was apparent and warranted quantitative investigation. Second, the *E*_mech, CM_ data set is limited in size and range compared to the *E*_met_ data. Clearly, parity of species in both mechanical and metabolic data should be maintained to avoid any bias. Accordingly, here the relationship between the *E*_mech, CM_ and *E*_met_ of transport and *M*_b_ was re-examined to determine whether a size dichotomy in locomotor performance exists. In addition, to prevent a data set size bias, only *E*_met_ data for species that were also included in the *E*_mech, CM_ data were used.

## Methods

The mass specific cost of locomotion (J m^−1^ kg^−1^), whether mechanical (*E*_mech, CM_) or metabolic (*E*_met_), is equal to the mass specific power (W kg^−1^) divided by the speed (m s^−1^). This is commonly calculated by plotting the mass specific power at a range of speeds and extracting the gradient of the best-fit straight line, and then using this value as the speed averaged mass specific cost of locomotion [3], [6], [7]. This method of calculation, however, assumes a model where there is a linear relationship between speed and power, and where the intercept of the best-fit straight line represents the additional energetic costs that an animal has, that are independent of the cost of locomotion. There is generally insufficient velocity resolution to test this model in available datasets but in horses, for example, where high quality data are available, it has been found to be false [8], [9]. In the case of mechanical power there is a good argument for enforcing an intercept of zero when calculating the line of best fit, since the centre of mass is static meaning there is no temporal change in kinetic or potential energy when the forward velocity is zero. This is not generally done, however, and, for example, the intercept values for mechanical power for the species studied by Cavagna et al. [6], and Heglund et al. [3] were different from zero. Furthermore, for some of the species, intercepts were negative suggesting a stationary animal would be gaining mechanical energy. For metabolic energy a better approach is to subtract the power required for standing from the power required during locomotion and then dividing by speed [10]. The minimum value obtained over a range of speeds is then used as the representative cost of locomotion. This is the approach generally used in the human literature but is not always possible to retrofit onto published data. Nonetheless, for species that do not fit the standard model it is necessary: for example using the slope value determined for a hopping Red Kangaroo (*Macropus rufus*), gives a zero or negative value for *E*_met_, because the slope is slightly negative [4], [11].

Here a pragmatic approach for obtaining values from the literature was used since in most cases it was not possible to directly recalculate the values, and the errors introduced by mixing values from different studies were thought likely to outweigh the benefits that could be obtained by using a better analytical approach. *E*_mech, CM_ data are mainly from Table 1 of Heglund *et al.* [3], with corrected values extracted from the original source [6], [7] and *E*_met_ from Table 1 of Taylor *et al.* [4]. Returning to the original source for some values was necessary, because the Cavagna *et al.* [6], [7] data included in Heglund *et al.* [3] was converted into S.I. units inaccurately. Unlike previous studies [1], [2], data were then averaged where a species occurred more than once (Table 1). For *Macropus rufus* a resting power was estimated from the intercept derived from the metabolic power versus speed equation for pentapedal locomotion (kangaroo low speed gait) [11] and was subtracted from the power value for high speed hopping. This was then divided by the maximum speed (6.11 m s^−1^) recorded in the original study by Dawson and Taylor [11] to give 0.1707 mlO_2_ m^−1^ kg^−1^.

**Table 1:** Data used in the analyses.

Energy type	Species	*n*	Body mass (kg)	J m^−1^ kg^−1^
***E*** **_met_**	*Dipodomys merriami*	3	0.03	56.35
	*Excalfactoria chinensis*	1	0.04	24.12
	*Tamias striatus*	2	0.08	23.72
	*Colinus virginianus*	1	0.19	18.09
	*Spermophilus tereticaudus*	1	0.24	13.27
	*Pedetes capensis*	1	3	6.83
	*Meleagris gallopavo*	1	4.31	8.24
	*Macaca speciosa*	1	5.1	5.03
	*Canis familiaris*	5	13.99	4.46
	*Rhea americana*	1	22	6.83
	*Macropus rufus*	1	23	3.43
	*Ovis aries*	1	23	4.62
	*Homo sapiens*	1	68.8	4.02
***E*** **_mech, CM_**	*Dipodomys merriami*	2	0.07	1.13
	*Excalfactoria chinensis*	1	0.04	1.68
	*Tamias striatus*	1	0.1	1.28
	*Colinus virginianus*	1	0.18	1.57
	*Spermophilus tereticaudus*	1	0.19	0.47
	*Pedetes capensis*	1	2.5	1.41
	*Meleagris gallopavo*	1	7	1.43
	*Macaca speciosa*	1	3.6	1.85
	*Canis familiaris*	1	11	0.94
	*Rhea americana*	1	22.5	1.00
	*Macropus rufus*	1	20.5	1.58
	*Ovis aries*	1	73	0.49
	*Homo sapiens*	1	70	1.19

Mechanical (*E*_mech, CM_) data are corrected data (see methods) from table 1 of Heglund et al. [3] and metabolic (*E*_met_) data are collated from table 1 of Taylor et al. [4], and Dawson, and Taylor [11].

In the original analysis of Full [2], *E*_mech, CM_ data for two invertebrate species (cockroach and ghost crab) were also included. These data points were omitted here, because they represented a third data set, a very different taxanomic group (invertebrates) and the small sample size (*n* = 2) is not sufficient to determine statistically valid patterns in data.

The first stage in the statistical analysis used an analysis of covariance (ANCOVA) to determine whether the relationship between the log_10_ cost of transport (*E*) and log_10_ *M*_b_ differed between *E*_met_ and *E*_mech, CM_ for all thirteen species grouped together (henceforth referred to as model A). The data were then split into two groups. The first group contained the five species with body masses <1 kg [3] and the second group species with body masses >1 kg [6], [7]. The relationship between *E*_met_ or *E*_mech, CM_ and *M*_b_ was then determined for these two different size groups (four groups of data in total i.e., *E*_met_ and *E*_mech, CM_ for animals of <1 kg, and *E*_met_ and *E*_mech, CM_ for animals>1 kg). ANCOVA was again used to establish whether differences existed in the relationship between *E* (*E*_met_ or *E*_mech, CM_) and *M*_b_ for the four different groups (henceforth model B). Akaike's Information Criteria corrected for a small sample size (AIC*_c_*) [12], [13] was used to determine which of the two ANCOVA models (A or B) best described the relationship between *E* (*E*_met_ and *E*_mech, CM_) and *M*_b_. Due to the small sample sizes, phylogenetically informed methods [14] were not employed. Analysing the data at the species level, however, is consistent with previous analyses [2]–[4] of the relationship between *E*_met_ and *E*_mech, CM_ and *M*_b_. All analyses were carried out using the statistics toolbox in MATLAB® R2007b (The MathWorks, Inc., 3 Apple Hill Drive, Natick, MA).

## Results

**Figure 1:**
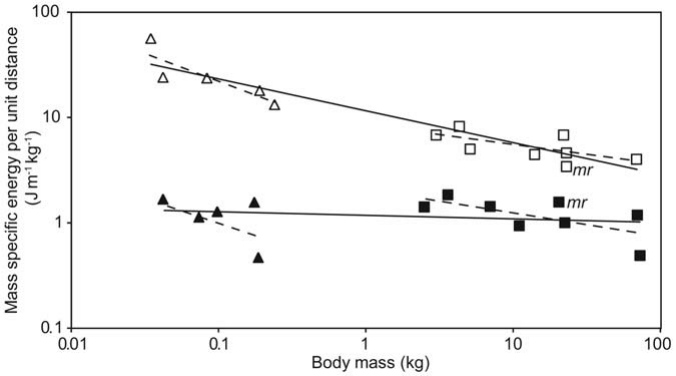
The relationship between the mass specific cost of transport and body mass. Open symbols represent metabolic energy (*E*_met_), closed symbols mechanical energy (*E*_mech, CM_), triangles are species with <1 kg body masses [3] and squares species>1 kg [6], [7]. Data for *Macropus rufus* are labelled with *mr* symbols. Individual trend-lines calculated from the ANCOVA coefficient tables are for the *E*_mech, CM_ data; *y* = 1.17*x* ^−0.03^ (all data grouped), *y* = 2.06*x* ^−0.22^ (species>1 kg) and *y* = 0.391*x* ^−0.46^ (species<1 kg). For the *E*_met_ data the trend-lines are *y* = 11.38*x* ^−0.30^, *y* = 8.38*x* ^−0.19^ and *y* = 6.55*x* ^−0.53^ for all data grouped, species>1 kg and species<1 kg respectively.

**Figure 2:**
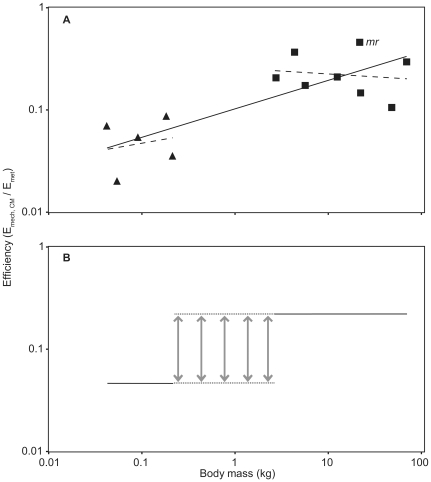
The scaling of efficiency (*E*_mech, CM_/*E*_met_) against body mass in terrestrial locomotion. (A) The scaling relationships for all data grouped (solid line) and for the two size classes (dashed lines). Triangles are species with <1 kg body masses [3] and squares species>1 kg [6], [7]. Data points for *Macropus rufus* are labelled with *mr*. Individual trend-lines are for all data grouped; *y* = 0.10*x* ^0.27 (0.13–0.41)^, *t* = 4.11, *n* = 13, *r* ^2^ = 0.61, *p*<0.05; species<1 kg, *y* = 0.07*x* ^0.16 (−1.30–1.613)^, *t* = 0.34, *n* = 5, *r* ^2^ = 0.04, *p*>0.05; species>1 kg, *y* = 0.26*x* ^−0.06 (−0.48–0.355)^, *t* = −0.36, *n* = 8, *r* ^2^ = 0.02, *p*>0.05. (B) Hypothetical step-change relationship between efficiency and body size. At some point within the size range depicted by the dotted lines efficiency may shift from 7% to 26%.

Using model A, both the intercept and slope describing the relationship between *E*_met_ and *M*_b_ differed from that between *E*_mech, CM_ and *M*_b_ (ANCOVA: *E* (intercepts), *F*_1, 22_ = 199.57, *p*<0.001; *M*_b_, *F*_1, 22_ = 33.96, *p*<0.001; *E* * *M*_b_ (slopes), *F*_1, 22_ = 22.99, *p*<0.001). *E*_mech, CM_ was independent of *M*_b_ (Fig. 1) when all data were grouped together and the mean *E*_mech, CM_ of 1.17 J m^−1^ kg^−1^ (Table 1) was 9% higher than the 1.07 J m^−1^ kg^−1^ previously determined by Full [2]. In contrast, *E*_met_ scaled predictably as *M*_b_^−0.30^, which was comparable to the exponent determined previously: *M*_b_^−0.32^ [2]. Therefore, using all thirteen species, efficiency (*E*_mech, CM_/*E*_met_) scaled positively with *M*_b_ (Fig. 2). Interestingly, however, using model B showed an apparent difference between the two *E*_mech, CM_ size groups (Fig. 1), with the scaling of *E*_met_ and *E*_mech, CM_ similar within each group. In fact, although the four intercepts for *E*_met_ and *E*_mech, CM_ for the two size groups differed, the slopes of the relationships between *E*_met_ and *E*_mech, CM_ and *M*_b_ did not (ANCOVA: group (intercepts), *F*_3, 18_ = 93.16, *p*<0.001; *M*_b_, *F*_1, 18_ = 14.17, *p* = 0.001; group * *M*_b_ (slopes), *F*_3, 18_ = 1.28, *p* = 0.409). The ANCOVA also showed that overall *E* (both *E*_met_ and *E*_mech, CM_) decreased significantly with *M*_b_. The *E*_met_ and *E*_mech, CM_ exponents (for animals<1 kg, *E*_met_ ∝ *M*_b_^−0.53^ and *E*_mech, CM_ ∝ *M*_b_^−0.46^_,_ and for animals>1 kg, *E*_met_ ∝ *M*_b_^−0.19^, and *E*_mech, CM_ ∝*M*_b_^−0.22^) exhibited a similar pattern to those found previously by Reilly et al. [5]. This second ANCOVA (model B) explained more of the variation in the data than model A above (*r* ^2^ = 0.94 versus 0.92). However, because no difference was found between the four slopes, the model B ANCOVA was simplified further by removing the group×*M*_b_ interaction term, meaning the four individual trend-lines were treated as parallel and not separate. The resulting ANCOVA (group (intercepts), *F*_3, 21_ = 92.97, *p*<0.001; *M*_b_, *F*_1, 21_ = 14.14, *p* = 0.001) indicated a common slope of *E* ∝ *M*_b_^−0.26^. Although, the *r* ^2^ of the simplified model B (0.93) was still higher than that of model A, it has fewer parameters than model A and therefore using *r* ^2^ values to compare them is misleading. Accordingly, Akaike's Information Criteria, which compensates for parameter number, was used to compare the two statistical models, and Model 2 (AIC_c_ = −27.67) was 12.2 times more likely to be correct than model 1 (AIC_c_ = −22.67).

Scaling of efficiency (*E*_mech, CM_/*E*_met_) in terrestrial locomotion also scaled against *M*_b_, if all data were grouped together (Fig. 2) suggesting 4% efficiency for the smallest animal here (0.03 kg, *Dipodomys merriami*) and 32% for the largest (73.00 kg, *Ovis aries*). In contrast, within each size group there was no detectable size dependent variation in efficiency (Fig. 2). The intercepts of the trend-lines, however, indicated that efficiency in small animals weighing less than <1 kg was lower (7%) than that of the >1 kg animal group (26%). The results were similar if the data for *Macropus rufus* were excluded.

## Discussion

Our analysis suggests that the relationship between *E*_mech, CM_ is different for small (<1 kg) and large animal groups (>1 kg). Both the mass specific metabolic energetic cost and mechanical cost of transport decreases with increasing *M*_b_ in both data sets signifying that there is no scaling of efficiency within each size range. The data do indicate that the scaling of efficiency of locomotion, however, is generally less in the smaller animals (Fig. 2) indicating a size dependent step-change (from 7 to 26%) in efficiency, rather than a continuous linear relationship between efficiency and *M*_b_. 7% efficiency is perhaps more convincing than the 0.6% previously estimated for the smallest animals [1]. Reilly et al. [5] showed that there are non-linear patterns in effective mechanical advantage, limb muscle mass, stride characteristics and metabolic cost between animals with crouched stances, and those with erect stances. The obvious division in the data set presented here is size, but although Reilly et al. [5] divided their data according to posture criteria, this also resulted in a size division along similar lines to the present study (i.e., animals<1 kg and animals>1 kg). Therefore, it is possible that the step-change in locomotor efficiency observed here is driven by posture. Size dependent physiological differences may also affect locomotor efficiencies. Higher ventilatory and heart rates, and, due to the surface area/volume scaling relationship, higher rates of heat loss in smaller animals could effect the conversion of *E*_met_ into *E*_mech, CM._ These physiological differences, however, vary continuously with body size and therefore, currently do not offer a satisfactory explanation for a step change in locomotor efficiency. Another factor that must be considered is that the *E*_mech, CM_ data for the small animals were gathered in a different study [3] to that of the larger animals [6], [7]. Despite the fact that with the exception of the force plates used methodologies were consistent across the studies [3], the paucity of *E*_mech, CM_ data, and the fact that the range of animal sizes was also split between separate studies, means that a non-biological explanation for the size dichotomy cannot be entirely ruled out.

Treating the thirteen species as a single homogenous data set produces the same results here as found previously [2]: *E*_met_ scales with *M*_b_ and *E*_mech, CM_ is invariant across animal sizes. Therefore, using a much smaller *E*_met_ data set than used by previous authors [2], which only included the species also incorporated in the *E*_mech, CM_ data set, has little or no effect. In contrast, a slight increase in the estimate of the mean *E*_mech, CM_ was seen. Nonetheless, the reduced *E*_met_ and *E*_mech, CM_ data sets used here appeared to be comparable to those used previously and the omission of the two insect species included by Full [2] from the *E*_mech, CM_ had no effect on the scaling exponent.

Very little data for *E*_mech, CM_ is available and the few studies [2], [15], [16] that have been conducted since the work of Heglund et al. [3] and Cavagna et al. [6], [7] are not comparable with the data analysed in this study because of inconsistent methods (e.g. force plates were not used). Furthermore, small sample sizes preclude the investigation of study or taxa effects. Consistent methodology is essential when comparing across studies, because previous work has shown that different calculation methods result in different estimates of *E*_mech, CM_ [17]–[19]. Pertinently, for the data analysed in this study, the use of force plates is thought to provide slightly less accurate measures of *E*_mech, CM_ than kinematic techniques (i.e., sacral marker and segmental analysis methods) utilising motion capture and inverse dynamics. Irrespective of the accuracy or otherwise of the force plate technique, however, the previous conclusion of mass dependent scaling of efficiency in locomotion [1], [2] is based upon it.

In conclusion, it appears that how efficiency scales with body size in terrestrial locomotion may not be a simple linear increase across animal sizes. Assuming there is no non-biological study effect, the limited data available does suggest smaller animals are generally less efficient in their locomotion than large animals, but within each size group no scaling of efficiency is evident. Therefore, there is an apparent step-change in the locomotor efficiencies of small and large animals. Alexander [1] suggested that the scaling of efficiency may also scale linearly with *M*_b_ in flight. The *E*_mech,_ data supporting this, however, are based upon theoretical models and scaling analyses [20] and not solely empirical data, and therefore the exact scaling pattern of efficiency in flight is yet to be determined. For swimming animals there is no evidence, either theoretical or empirical, for size dependent scaling of locomotor efficiency [1]. Consequently, the exact nature of size dependent scaling of efficiency in any form of locomotion is far from certain, but this current study and the work of Reilly et al. [5] suggests that it may be more complex than previously thought, at least in terrestrial locomotion. Homogenous data across a broad range of species, locomotor modes and body sizes, remains a priority for future research into the *E*_mech, CM_ of all forms of locomotion.

## References

[1] Alexander RM (2005). Models and the scaling of energy costs for locomotion.. J Exp Biol.

[2] Full RJ, Wieser W, Gnaiger E (1989). Mechanics and energetics of terrestrial locomotion: bipeds to polypeds.. Energy transformations in cells and organisms.

[3] Heglund NC, Cavagna GA, Taylor CR (1982). Energetics and mechanics of terrestrial locomotion III. Energy changes of the center of mass as a function of speed and body size in birds and mammals.. J Exp Biol.

[4] Taylor CR, Heglund NC, Maloiy GMO (1982). Energetics and mechanics of terrestrial locomotion I. Metabolic energy-consumption as a function of speed and body size in birds and mammals.. J Exp Biol.

[5] Reilly SM, McElroy EJ, Biknevicius AR (2007). Posture, gait and the ecological relevance of locomotor costs and energy-saving mechanisms in tetrapods.. Zoology.

[6] Cavagna GA, Heglund NC, Taylor CR (1977). Mechanical work in terrestrial locomotion: two basic mechanisms for minimizing energy-expenditure.. Am J Physiol.

[7] Cavagna GA, Thys H, Zamboni A (1976). Sources of external work in level walking and running.. J Physiol Lond.

[8] Hoyt DF, Taylor CR (1981). Gait and the energetics of locomotion in horses.. Nature.

[9] Minetti AE, ArdigO LP, Reinach E, Saibene F (1999). The relationship between mechanical work and energy expenditure of locomotion in horses.. J Exp Biol.

[10] Kramer PA, Sarton-Miller I (2008). The energetics of human walking: Is Froude number (Fr) useful for metabolic comparisons?. Gait & Posture.

[11] Dawson TJ, Taylor CR (1973). Energetic cost of locomotion in kangaroos.. Nature.

[12] Akaike H (1974). A new look at statistical-model identification.. IEEE Trans Automat Contr.

[13] Burnham KP, Anderson DR (2001). Kullback-Leibler information as a basis for strong inference in ecological studies.. Wildlife Research.

[14] Felsenstein J (1985). Phylogenies and the comparative method.. Am Nat.

[15] Reilly SM, McElroy EJ, Odum RA, Hornyak VA (2006). Tuataras and salamanders show that walking and running mechanics are ancient features of tetrapod locomotion.. Proc R Soc Lond B.

[16] Pfau T, Witte TH, Wilson AM (2006). Centre of mass movement and mechanical energy fluctuation during gallop locomotion in the Thoroughbred racehorse.. J Exp Biol.

[17] Arampatzis A, Knicker A, Metzler V, Bruggemann GP (2000). Mechanical power in running: a comparison of different approaches.. J Biomech.

[18] Saini M, Kerrigan DC, Thirunarayan MA, Duff-Raffaele M (1998). The vertical displacement of the center of mass during walking: A comparison of four measurement methods.. J Biomech Eng-Trans ASME.

[19] Thirunarayan MA, Kerrigan DC, Rabuffetti M, Croce UD, Saini M (1996). Comparison of three methods for estimating vertical displacement of center of mass during level walking in patients.. Gait & Posture.

[20] Rayner JMV (1995). Flight mechanics and constraints on flight performance.. Isr J Zool.

